# Life Cycle Assessment of Innovative Carbon Dioxide Selective Membranes from Low Carbon Emission Sources: A Comparative Study

**DOI:** 10.3390/membranes13040410

**Published:** 2023-04-05

**Authors:** Amit S. Nilkar, Christopher J. Orme, John R. Klaehn, Haiyan Zhao, Birendra Adhikari

**Affiliations:** 1 Chemical Separations Group, Material Separation and Analysis Department, Idaho National Laboratory (INL), Idaho Falls, ID 83415, USAjohn.klaehn@inl.gov (J.R.K.); 2 Department of Chemical and Biological Engineering, The University of Idaho, Moscow, ID 83844, USA

**Keywords:** life cycle assessment, global warming, acidification, carbon capture and utilization, fossil fuel depletion, membrane separation

## Abstract

Carbon capture has been an important topic of the twenty-first century because of the elevating carbon dioxide (CO_2_) levels in the atmosphere. CO_2_ in the atmosphere is above 420 parts per million (ppm) as of 2022, 70 ppm higher than 50 years ago. Carbon capture research and development has mostly been centered around higher concentration flue gas streams. For example, flue gas streams from steel and cement industries have been largely ignored due to lower associated CO_2_ concentrations and higher capture and processing costs. Capture technologies such as solvent-based, adsorption-based, cryogenic distillation, and pressure-swing adsorption are under research, but many suffer from higher costs and life cycle impacts. Membrane-based capture processes are considered cost-effective and environmentally friendly alternatives. Over the past three decades, our research group at Idaho National Laboratory has led the development of several polyphosphazene polymer chemistries and has demonstrated their selectivity for CO_2_ over nitrogen (N_2_). Poly[bis((2-methoxyethoxy)ethoxy)phosphazene] (MEEP) has shown the highest selectivity. A comprehensive life cycle assessment (LCA) was performed to determine the life cycle feasibility of the MEEP polymer material compared to other CO_2_-selective membranes and separation processes. The MEEP-based membrane processes emit at least 42% less equivalent CO_2_ than Pebax-based membrane processes. Similarly, MEEP-based membrane processes produce 34–72% less CO_2_ than conventional separation processes. In all studied categories, MEEP-based membranes report lower emissions than Pebax-based membranes and conventional separation processes.

## 1. Introduction

Climate change has been identified as the most serious environmental threat to human life in the twenty-first century [1]. Climate change is so severe that many big cities developed along the coastline can potentially be underwater in the near future. The risks of flooding have increased simultaneously with the occurrence of historical droughts [2]. Mountains are seeing less snow and an increased frequency of avalanches [3]. Snowcaps are melting in polar regions at an unprecedented rate, threatening marine ecosystems [4].

Among the many causes of global warming, the unprecedentedly high carbon dioxide (CO_2_) level in the atmosphere is considered the most important cause [5,6]. The level of CO_2_ has increased by 70 parts per million (ppm) in the air during the last 50 years and by more than 50% since the pre-industrial era [7]. This increase in atmospheric CO_2_ matches CO_2_ emissions, which have gone up by more than 90% since 1970 [8]. Far more CO_2_ is emitted into the atmosphere than what is consumed by photosynthesis or other storage processes [9]. The formation of natural oil and gas takes millions of years but will be consumed in a hundred years at the current pace. The burning of natural gas and oil produces CO_2_ that goes directly into the atmosphere. Devices associated with daily life also contain embedded CO_2_ emissions [10]. Indirect emissions of CO_2_ are measured by the manufacturing, transportation, and use of those items throughout their lifetime.

Human daily activities can not be paused. Energy sources such as electricity, heat, and gasoline drive our daily activities. Energy is needed for transportation, household life, and many other activities. Every kilowatt-hour (kWh) of electrical energy use, every gallon of gasoline burnt, or every therm of heat generated has an equivalent associated CO_2_ emission [11,12]. This means that CO_2_ will be emitted continuously as long as energy is consumed. While pausing energy consumption is not an option, capturing emitted CO_2_ can achieve a carbon-based sustainable circular economy. The captured CO_2_ can be either reclaimed to make more energy by converting it to fuels and chemicals [5,13,14], used as a medium to do other activities such as enhanced oil recovery, or stored in a stable form, as done in mineralization [15,16,17,18].

Potential methods for capturing CO_2_ have been identified; however, it has yet to be widely adopted and commercialized [19]. Chemical absorption into liquid solvents from gas streams, at present, is the most widely accepted form of capturing CO_2_ [20]. However, the cost, reusability, and life cycle impacts of solvent-based processes make them unattractive [21]. In addition, solvent-based processes often require significant processing equipment, where the cost and life cycle impacts of the capital equipment alone are very high [22]. This has prompted researchers to lean towards more cost-effective and environmentally sustainable carbon capture alternatives. Technologies such as pressure swing adsorption [23] and cryogenic distillations [24] are also considered alternatives to solvent-based capture but have high capital and energy cost, thus making them environmentally unsustainable [25,26]. Membranes are usually more environmentally friendly than other technologies [27,28,29,30,31]. Membranes are known to have lower environmental footprints, and since no solvents are being used in most membrane technologies, their operating cost is lower [32]. However, the capital cost is usually higher for membrane processes [33]. In comparison to other processes, product purity of membrane processes is often lower, thus needing further processing [34]. Membranes have been considered for carbon capture and utilization recently due to their advantages over other systems due to lower carbon footprint, process simplicity, low production cost, ease of operation, compactness, and scale-up feasibility [35]. Membranes with high CO_2_ permeance and CO_2_/N_2_ selectivity possess great potential to capture CO_2_ from any feed gas, including air; however, most membrane technologies still need to be thoroughly investigated for this application [36].

Direct air capture (DAC) has been increasingly become a focus of research in recent years as a means of capturing CO_2_ from the air. State-of-art DAC processes operate by sucking in air through large fans, which is treated with a solid or liquid sorbent and then heated to extract CO_2_ [37]. However, the concentration of CO_2_ is very small (approximately 420 ppm) compared to flue gases from industrial carbon sources, making the processing volume unsustainably high [38,39].

**Figure 1:**
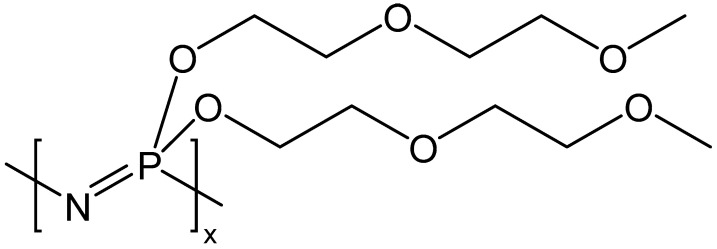
Chemical structure of MEEP.

Low-concentration carbon capture, such as exhaust gases from industries such as cement and steel [40], has been ignored because of the high cost of capture and high environmental footprints [41]. Membranes give the best and most cost-effective separation options [42,43,44,45,46]. Our research group has developed membranes that can enrich CO_2_ concentration from low carbon emitting sources using the concept of low-concentration CO_2_ enrichment [47]. Over the course of last three decades, several unique polyphosphazenes have been developed for a variety of applications [43,44,45,46,48,49,50,51,52,53,54,55,56,57,58]. Among these polyphosphazenes, poly[bis((2-methoxyethoxy)ethoxy) phosphazene (MEEP, Figure 1) is a specialty polymer that can fabricate thin, self-healing CO_2_/N_2_ selective membranes [59]. These polymers tend to have flexible chains that permit fast gas transport and gas solubility difference for CO_2_ and N_2,_ and because of this special trait, have shown excellent CO_2_/N_2_ selectivity and CO_2_ permeability. Furthermore, these polymers can be made extremely thin so that the cost of production can be minimized. In addition, this polymer material is durable and can be blended with other materials, such as C18 functionalized nanodiamonds, to enhance mechanical properties [28,60].

For CO_2_/N_2_ separation, Pebax elastomers from Arkema are used to develop state-of-art membranes with high CO_2_/N_2_ selectivity and CO_2_ permeability. Emerging CO_2_ selective membranes are always compared against pristine Pebax or Pebax derivatives. To quantify the environmental advantages of these membranes over other membrane and separation technologies, a detailed life cycle assessment (LCA) is necessary. A detailed life cycle assessment is performed and reported in this manuscript using the United States Department of Energy (US DOE) developed Greenhouse gases, Regulated Emissions and Energy in Transportation model and database, commonly known as GREET. Techno-economic analysis (TEA) results are reported separately (see Supplementary). The basis of CO_2_ separation is 1% CO_2_ in nitrogen for MEEP membranes, while the basis of separation parameters for other membranes is reported in the literature and illustrated in Table 1. Most membranes have used pure gas measurements and some have used 50/50 CO_2_/N_2_ mix.

## 2. Methods

### 2.1. Goal and Scope

LCA is a recent methodical approach gaining popularity over the last three decades. It is a methodology used to analyze a product’s environmental impact at every stage of its “life”, from start to finish, often referred to as “cradle to grave” [62]. LCA calculates environmental releases corresponding to a product, starting with the extraction of raw materials, manufacturing, transport, and the use and disposal of the product. LCA is a valuable and powerful tool in determining the effects caused by a product and the production process of that product and whether it does more harm to the environment than good. Our analysis assumes that the energy extracted and produced is in the United States. The functional unit of our study is 1 kg of CO_2_ produced in CO_2_ enrichment; CO_2_ is the final product. The database was obtained from GREET, a fuel-cycle model that Argonne National Laboratory of US DOE developed. The model calculates fuel cycle emissions of six pollutants: carbon monoxide, nitrogen oxides, sulfur oxides, volatile organic compounds, and particulate matter with a diameter of 10 microns or less, and three greenhouse gases: CO_2_, methane, and nitrous oxide [63]. The model is also used to calculate the effects of different energy sources, emissions of different transportation technologies, and the different assumptions used to reach their respective conclusions. The values were directly extracted from the GREET database and displayed on an Excel spreadsheet, which was used for the subsequent life cycle inventory analysis (LCIA). From the LCIA, the total impact of the system was calculated based on four impact categories. The impact categories that were used in this study are: global warming potential (GWP), acidification potential (AP), respiratory effects (RE), and fossil fuel depletion (FFDP). GWP refers to potential of global warming caused by greenhouse gas emissions into the air by gases such as CO_2_, methane, and nitrous oxide. Each has its potency for CO_2_. For example, methane has been determined to be 25 times more potent than CO_2_ for its effect on climate change [64]. This impact category is measured in kg of CO_2_ equivalent per functional unit. RE is a measure of the amount of particulate matter smaller than 2.5 microns or less in width emitted from the process that can cause respiratory problems. It is measured in kg of PM 2.5 equivalent per functional unit. AP is an evaluation of the amount of sulfur dioxide (SO_2_) emissions caused from the process. It is measured in kg of SO_2_ equivalent per functional unit. FFDP is an indicator of the amount of fossil fuel the process consumes. It is measured in megajoules (MJ) per functional unit. These were the impact categories that could be determined from the data obtained from GREET. Associated results and sensitivity analysis are completed to understand the overall life cycle aspects of the membrane processes [65].

For a process to be sustainable, it has to be economically and environmentally sustainable [66,67,68,69,70,71,72]. Comparative LCA is performed for different electricity mixes, including the current US Mix, capital equipment and membrane materials involved in producing the enriched gas products [73,74]. Our study focuses on the comparison of the life cycle emission of two different INL gas separation membrane processes: (i) MEEP-only membrane (MEEP membrane) and (ii) MEEP with C18 functionalized nanodiamonds (MEEP/CND membrane), respectively, and several Pebax-derived membranes including pristine Pebax membranes (Pebax lower end (Pebax LE) membrane and Pebax higher end (Pebax HE)), and nanofillers filled membranes, such as Pebax ZIF-8. Membrane processes with one stage (single-staged), two stages (double-staged), and three stages (triple-staged) processes have been considered and analyzed.

### 2.2. Processes Studied

In this study, membranes are evaluated using several known LCA categories. Our study covers the LCA of a set of 15 commercial membranes in three different-staged scenarios along with two INL membranes. It compares the greenhouse gas emissions and other environmental feasibility categories using the GREET model and database. The basis of CO_2_ separation is 1% CO_2_ in nitrogen (1%) while the basis of separation parameters obtained for other membranes is as reported in the literature and illustrated in Table 1. Most membranes have used pure gas measurements and some have used 50/50 CO_2_/N_2_ mix.

### 2.3. System Boundary

**Figure 2:**
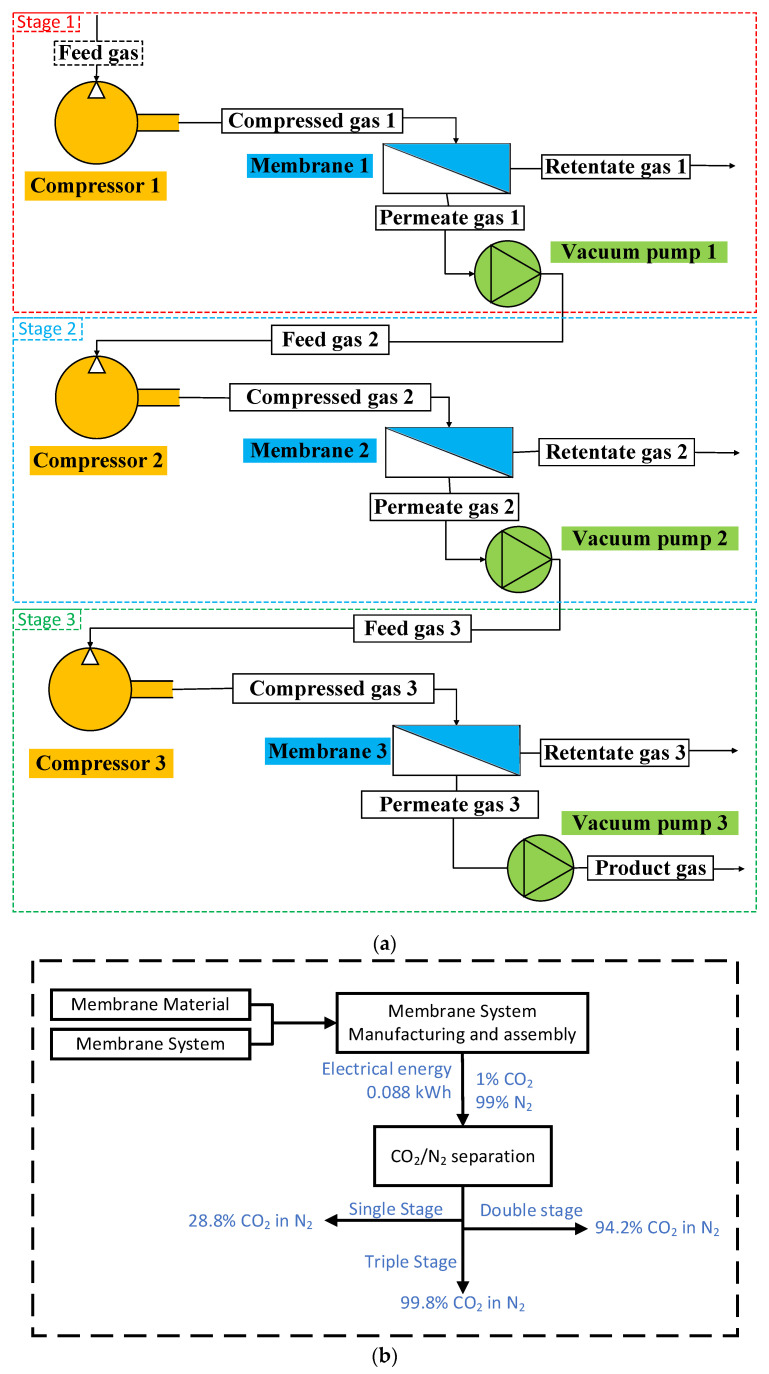
(**a**) Process flow diagram (PFD) of CO_2_ enrichment system and (**b**) system boundary for the life cycle analysis.

The CO_2_ enrichment system consists of three membrane systems in series, as shown in Figure 2. A vacuum pump is installed on the permeate side of the membrane to facilitate mass transport, a common technique in industrial-scale processes. The pump size, the first gas separation unit, and the vacuum component are all dependent upon the amount of feed gas that is being supplied, the selectivity of CO_2_ and N_2_, and the CO_2_ permeability of the membrane. Following the first system, the membrane systems, including the compressor and vacuum components, are optional and chosen according to the process design. A second gas separation system follows the first unit in sequence, where the permeate of the first system becomes the feed to the second. A third unit is also considered and operated similarly in the proposed model. Figure 2a depicts the process flow diagram of triple staged membrane system:

### 2.4. Life Cycle Inventory

Table 2 summarizes the material and energy inputs to the membrane system. The life cycle inventory (LCI) includes the activities to produce 1 kg of CO_2_ for each kg of CO_2_ the system avoids. The inputs are the same, irrespective of how many membranes are used in the system. The unit processes for different input materials used in excel to analyze the environmental impacts are summarized in Table 2.

The membrane system and modules are made from stainless steel material, and membrane materials are assumed to be equivalent to polypropylene material.

### 2.5. Impact Assessment

This study uses GREET-defined impact categories: global warming potential, acidification potential, respiratory effects, and fossil fuel depletion potential.

## 3. Results and Discussion

### 3.1. Life Cycle Assessment: Single-Stage Process

**Figure 3:**
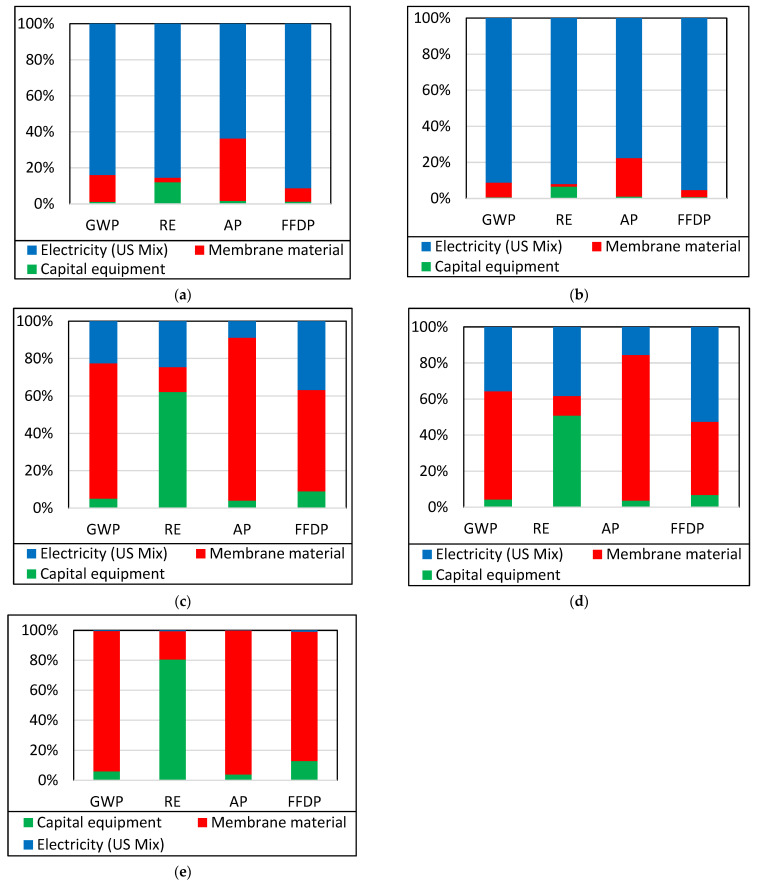
Analysis of contributions of the single-stage CO_2_ enrichment process with (**a**) MEEP, (**b**) MEEP/CN, (**c**) Pebax LE, (**d**) Pebax HE, and (**e**) Pebax ZIF-8 membranes.

A single-stage membrane process is considered to enrich 1% CO_2_ in N_2_ to 28% CO_2_ in N_2_. This purity is achieved by recovering 90% of CO_2_ present in the feed gas in the permeate. The membrane area and number of membrane modules needed for the process were calculated based on CO_2_/N_2_ selectivity, CO_2_ permeability, membrane thickness and operating parameters such as compressor pressure and vacuum pressure. The mass allocation method was used to evaluate the environmental impacts of producing 1 kg of CO_2_ per kg of CO_2_ avoided by the membrane. For a better understanding, Figure 3 shows the contribution of each component to the total environmental impact of a single-stage membrane process with MEEP, MEEP/CN, Pebax LE, Pebax HE and Pebax ZIF-8 membranes. The percentage contribution of each input is compared using a 100% stacked column bar chart. It is clear from the chart that capital equipment, membrane material, and energy used in the membrane processes are significant in each process studied. While comparing two MEEP-based INL membranes against three Pebax-based membranes in four major environmental impact categories, it is found that for MEEP-based INL membranes, electricity has the highest impact in all the categories studied. Membrane materials have the highest impact on Pebax-based membranes in GWP, AP, and FFDP categories, while capital equipment has the highest impact in the RE category. This indicates that MEEP-based INL membranes use less membrane material and capital equipment, making electricity the highest contributor. Pebax-based membranes use a high amount of capital equipment and membrane material, and as a result, electricity becomes a lesser contributor. Pebax ZIF-8 membrane material has the highest membrane material contribution.

### 3.2. Life Cycle Assessment: Two-Stage Process

**Figure 4:**
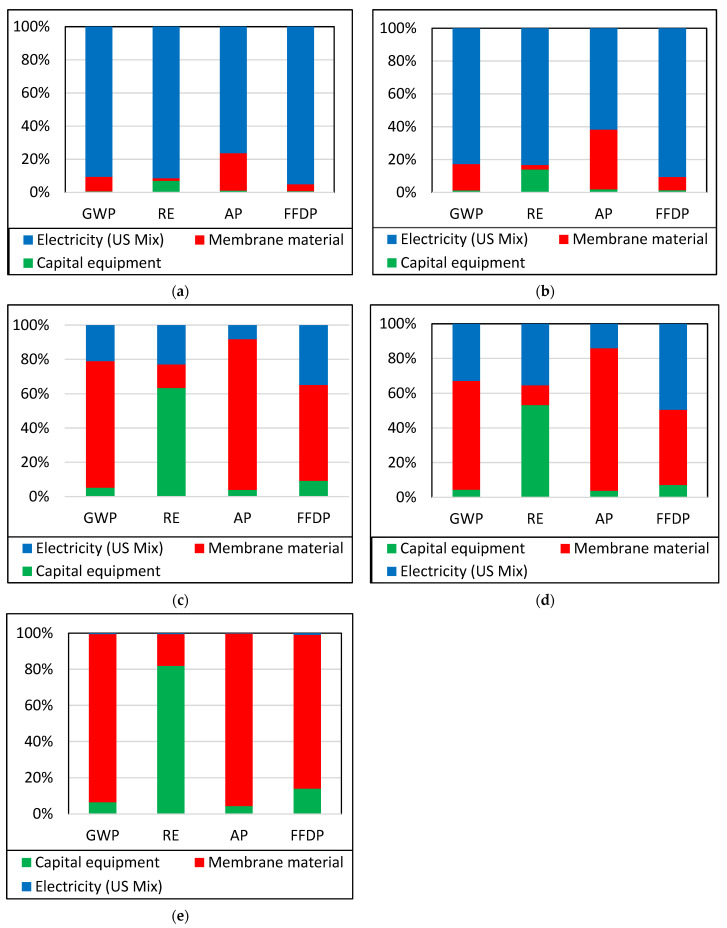
Analysis of contributions of the two-stage CO_2_ enrichment process with (**a**) MEEP, (**b**) MEEP/CN, (**c**) Pebax LE, (**d**) Pebax HE, and (**e**) Pebax ZIF-8 membranes.

Figure 4a,e show the percentage contribution of each of the five membranes in a two-stage process using a 100% stacked column bar chart. In this scenario, CO_2_ is enriched from 1% in N_2_ to 94% CO_2_ in N_2_ in two stages. Each stage has a 90% recovery of CO_2_. Other parameters are the same as in the single-stage system.

Membrane material and capital equipment contribute the most significant environmental impact for three Pebax-based membranes, but electricity still contributes the most environmental impact for MEEP-based INL membranes. This trend is similar to that seen in the single-stage process, indicating that MEEP-based membrane processes use far less membrane materials and capital equipment than the Pebax-based membrane processes.

### 3.3. Life Cycle Assessment: Three-Stage Process

**Figure 5:**
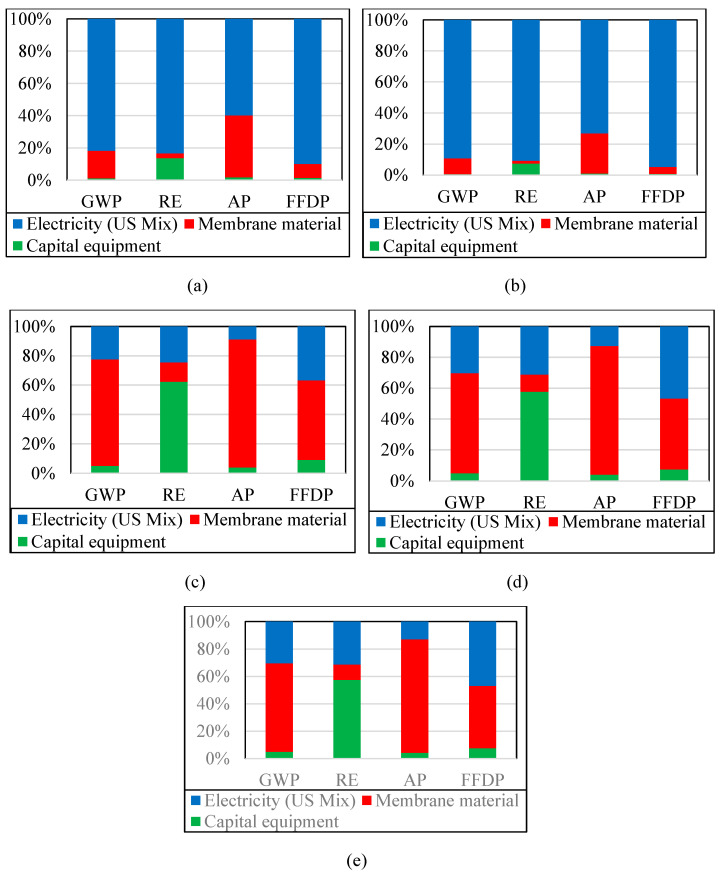
Analysis of contributions of the three-stage CO_2_ enrichment process with (**a**) MEEP, (**b**) MEEP/CN, (**c**) Pebax LE, (**d**) Pebax HE, and (**e**) Pebax ZIF-8 membranes.

Figure 5a,e show the percentage contribution of each of the five membranes in a three-stage process using a 100% stacked column bar chart. CO_2_ is enriched from 1% CO_2_ in N_2_ to >99% CO_2_ in N_2_ using three stages and 90% CO_2_ is recovered in each stage. All other parameters are kept constant as in single-stage and two-stage membrane processes.

Figure 5c–e indicate that membrane material and capital equipment contribute the highest impact amongst the three Pebax-based membranes, compared to the two MEEP-based INL membranes (Figure 5a,b), where electricity contributes the highest environmental impacts. This trend is like those in the single- and two-stage membrane processes. This suggests that MEEP-based INL membranes perform relatively consistently at varied purity levels. Capital equipment requirements drive membrane-based carbon capture processes, while MEEP-based capture processes are driven by electricity consumption from the standpoint of life cycle impact. 

### 3.4. Comparison to Other Membrane Processes

MEEP-based INL membranes were compared against each Pebax-based membrane, as illustrated in Table 3. These results represent a single-stage separation system with CO_2_ enriched from 1% CO_2_ in N_2_ to 28% CO_2_ in N_2_ in one stage. The separation properties of these membranes are given in Table 1. Four impact categories are computed for each membrane and compared against one another. Among all the analyzed membranes, MEEP and MEEP/CN membranes have the lowest impacts in all categories. Alone, the MEEP membrane has GWP emissions of 4.40 × 10^−2^ kg CO_2_ eq/kg CO_2_ avoided, and the MEEP/CN membrane has GWP emissions of 4.42 × 10^−2^ kg CO_2_ eq/kg CO_2_ avoided. State-of-the-art membranes Pebax LE and Pebax HE membranes have GWP emissions of 0.163 kg CO_2_ eq/kg CO_2_ avoided and 7.53 × 10^−2^ kg CO_2_ eq/kg CO_2_ avoided, respectively. This suggests that from GWP standpoint, MEEP membranes show a 73% performance improvement over the Pebax LE membrane and a 42% performance improvement over the Pebax HE membrane. In all other categories, MEEP and MEEP/CN membranes perform 50% better than Pebax LE and Pebax HE membranes and outperform all other membranes.

### 3.5. Comparison to Other Separation Processes

**Figure 6:**
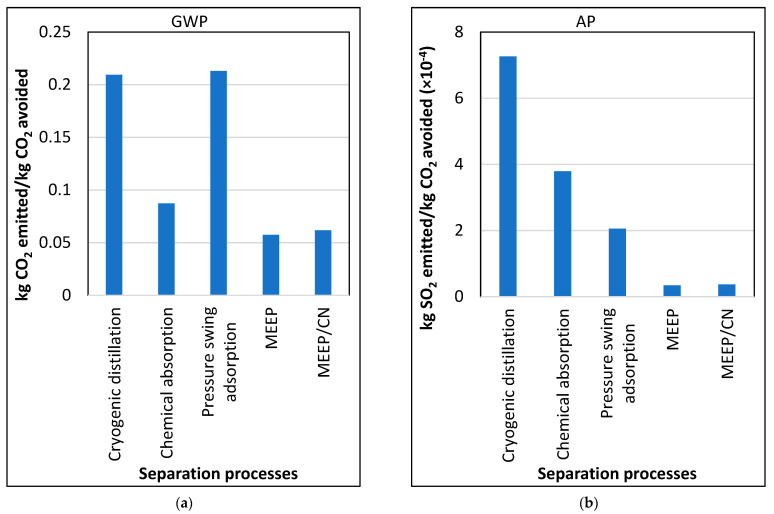
(**a**) Comparison of GWP of CCUs. (**b**) Comparison of AP of CCUs.

In this section, other major technologies are compared against MEEP and MEEP/CN membrane processes. Given the complexity of each process, GWP and AP for energy use for each separation process were compared. It is important to understand that most of the separation processes are dominated by capital equipment costs while MEEP and MEEP/CN processes are driven by electrical energy demands. Khoo et. al. compared chemical absorption, generic membrane separation, cryogenic distillation, and pressure swing adsorption in terms of equivalent CO_2_ emission per 950 kg of CO_2_ captured with varying efficiencies [75]. Those results were compared with the MEEP-based membrane process by converting units to kg CO_2_ emitted per kg of CO_2_ avoided. Regarding GWP, chemical absorption, cryogenic distillation, and pressure swing adsorption processes emit 0.087, 0.209 and 0.213 kg CO_2_ per kg of CO_2_ avoided, respectively. This suggests that the MEEP-based membrane process emits 34%, 72%, and 72% less CO_2_ than chemical absorption, cryogenic distillation, and pressure swing adsorption processes, respectively. For AP, cryogenic distillation, chemical absorption, and pressure swing adsorption emit 7.26 × 10^−4^, 3.79 × 10^−4^ and 2.06 × 10^−4^ kg SO_2_ per kg of CO_2_ emitted, respectively. This implies that the MEEP-based separation process emits 95%, 91%, and 83% less SO_2_ than cryogenic distillation, chemical absorption, and pressure swing adsorption separation processes, respectively. The following Figure 6 summarizes carbon capture and utilization (CCU) comparisons.

### 3.6. Sensitivity Analysis ᷇

A sensitivity analysis was conducted and tornado charts were plotted based on a 10% change in the inputs to the system in terms of GWP [65]. This analysis helps to identify the most sensitive input for a specific impact category. The five membranes considered for the staging evaluations were again considered for sensitivity analysis. A single-stage membrane process is the basis of sensitivity analysis.

For the MEEP membrane process, a 10% increase in electricity and membrane material demands resulted in an 8.42% and 1.47% increase in GWP, respectively. However, a 10% increase in capital equipment requirements resulted in an increase of 0.1% in GWP. For the MEEP/CN membrane single-stage process, a 10% increase in electricity demand resulted in a 9.14% increase in GWP. Similarly, a 10% increase in membrane material and capital equipment requirements resulted in only 0.81% and 0.05% increases in GWP, respectively. This indicates that the electricity required to drive the process is the most significant factor affecting the MEEP-based membrane process.

**Figure 7:**
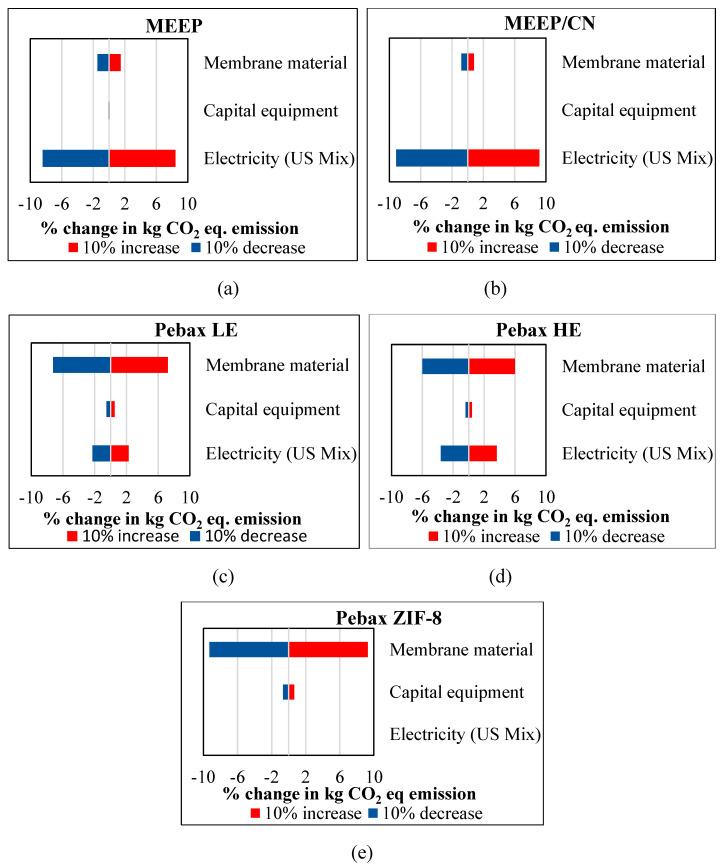
Sensitivity analysis of GWP by increasing and decreasing by 10% membrane material, capital equipment, and electricity requirements of (**a**) MEEP, (**b**) MEEP/CN, (**c**) Pebax LE, (**d**) Pebax HE, and (**e**) Pebax ZIF-8 membrane processes.

For the Pebax LE membrane process, a 10% increase in membrane material requirements resulted in a 7.22% increase in GWP, while a 10% increase in electricity consumption resulted in a 2.27% increase in GWP. A 10% increase in capital equipment requirements resulted in an increase in GWP of 0.51%. For the Pebax HE membrane process, a 10% increase in electricity and membrane material demands resulted in a 3.61% and 5.98% increase in GWP, respectively. However, a 10% increase in capital equipment requirements resulted in only a 0.42% increase in GWP. Similarly, for the Pebax ZIF-8 single stage process, a 10% increase in membrane material requirements resulted in a 9.30% increase; a 10% increase in capital equipment and electricity requirements resulted in only a 0.65% and 0.05% increase in GWP, respectively. This once again indicates that for MEEP-based membrane processes, electricity is the most dominant factor. In contrast, for Pebax-based membrane processes, the membrane material is the most dominant factor from the standpoint of GWP emissions. Figure 7 summarizes these results.

## 4. Conclusions

A comprehensive LCA of gas separation processes was performed using the GREET database by developing a Microsoft Excel-based model. Multiple scenarios of the CO_2_/N_2_ separation using Pebax-based membranes were compared with MEEP-based INL membranes in single-stage, two-stage, and three-stage processes. The results obtained in this study suggest that the MEEP-based INL membranes outperform Pebax-based membranes in all studied categories. The most significant contributor to GWP and all other LCA metrics for the Pebax-based membrane processes is the membrane material utilized. For the MEEP-based INL membrane processes, electrical energy consumption is the most significant contributor to GWP. The MEEP-based membrane processes emit at least 42% less equivalent CO_2_ than the best-performing Pebax-based membrane process. Similarly, MEEP-based membrane processes produce 34–72% less CO_2_ than conventional state-of-the-art separation processes such as cryogenic distillation, pressure swing adsorption, and chemical absorption. The two MEEP-based membranes emit lower emissions in all major categories studied than all Pebax-based membranes and other state-of-the-art conventional CO_2_ separation processes.

## Figures and Tables

**Table 1:** Membranes studied in this manuscript [61].

Membrane	Membrane Thickness (µm)	Feed Gas (CO_2_ vol%/N_2_ vol%)	Separation Condition (Temperature °C/Pressure Bar)	Permeability (Barrer)	CO_2_/N_2_ Selectivity
Pebax LE	1	pure gas	25/1	55.0	40.0
Pebax HE	1	pure gas	25/1	100.0	70.0
Pebax/ZIF-8	105	pure gas	23/1	105.0	34.8
Pebax/ZIF-8(90 nm)	55	pure gas	25/1	154.0	40.5
Pebax/ZIF-8–90(50)	75	pure gas	35/--	217.5	54.1
Pebax/NH2-ZIF-8	-	pure gas	25/1	163.8	62.0
Pebax/UiO-66	18	50/50	25/3	97.2	56.6
Pebax/NH2-MIL-53	75	pure gas	35/10	120.0	55.5
Pebax/MoS2 nanosheet	28	pure gas	30/1	52.3	90.6
Pebax/NaY	23	pure gas	30/2	82.8	35.0
Pebax/NOTT300	38	pure gas	25/10	395.2	61.2
Pebax/MCM-41	88	pure gas	25/2	122.5	53.0
Pebax/GO	83	20/80	35/2	105.0	41.2
Pebax/aminosilane-GO	83	20/80	35/2	166.3	45.2
Pebax/PEI-ZIF-8	1	50/50	25/1	13.0	49.0
MEEP	0.1	99/1	15/1	100.0	40.0
MEEP/CND	0.1	99/1	15/1	100.0	35.0

**Table 2:** LCI data to produce 1 kWh of electricity using US Mix, capital equipment, and membrane material.

Input Parameters	CO_2_ (kg)	CH_4_ (g)	N_2_O (g)	PM2.5 (mg)	SO_2_ (g)	Fossil Fuel Depletion (MJ)
US Mix (electricity)	0.390	0.854	0.008	24.5	0.247	5.46
Capital equipment	0.792	1.88	0.017	563.1	0.997	12
Membrane material	1.55	21.4	33.6	120.8	22.1	73

**Table 3:** Comparison of environmental impacts for different membranes.

Impact Category	Global Warming	Respiratory Effects	Acidification Potential	Fossil Fuel Depletion
Unit	kg CO_2_ eq/kg CO_2_ avoided	kg PM2.5 eq/kg CO_2_ avoided	kg SO_2_ eq/kg CO_2_ avoided	MJ surplus/kg CO_2_ avoided
MEEP only	4.40 × 10^−2^	5.22 × 10^−6^	5.70 × 10^−5^	0.566
MEEP/CN	4.42 × 10^−2^	5.48 × 10^−6^	5.55 × 10^−5^	0.592
Pebax LE	0.163	1.15 × 10^−5^	2.69 × 10^−4^	1.35
Pebax HE	7.53 × 10^−2^	6.10 × 10^−6^	1.19 × 10^−4^	0.698
Pebax/ZIF-8	8.42	4.18 × 10^−4^	1.5 × 10^−2^	55.3
Pebax/ZIF-8 (90 nm)	2.49	1.35 × 10^−4^	4.42 × 10^−4^	1.66
Pebax/ZIF-9 90 (50)	1.98	1.07 × 10^−4^	3.51 × 10^−3^	13.2
Pebax/NH_2_-ZIF-8	6.04 × 10^−2^	5.45 × 10^−6^	9.12 × 10^−5^	0.613
Pebax/UiO-66	1.04	5.77 × 10^−5^	1.85 × 10^−3^	7.1
Pebax/NH_2_-MIL-53	3.5	1.88 × 10^−4^	6.23 × 10^−3^	23.2
Pebax/MoS_2_ nanosheet	2.26	1.21 × 10^−4^	4.01 × 10^−3^	15.0
Pebax/NaY	2.16	1.17 × 10^−4^	3.83 × 10^−3^	14.5
Pebax/NOTT300	0.531	3.04 × 10^−5^	9.30 × 10^−4^	3.71
Pebax/MCM-41	4.14	2.21 × 10^−4^	7.37 × 10^−3^	37.5
Pebax/GO	5.4	2.89 × 10^−4^	9.61 × 10^−3^	35.8
Pebax/aminosilane-GO	3.21	1.72 × 10^−4^	5.71 × 10^−3^	21.4
Pebax/PEI-ZIF-8	0.496	2.88 × 10^−5^	8.66 × 10^−4^	3.51

## Data Availability

The data presented in this study are available on request from the corresponding author.

## Supplementary Materials

The following supporting information can be downloaded at: https://www.mdpi.com/article/10.3390/membranes13040410/s1, Table S1: Scenario inputs for the analysis.
