# Supplementary material for: Life Cycle Assessment of Innovative Carbon Dioxide Selective Membranes from Low Carbon Emission Sources: A Comparative Study
Source: Membranes (Basel). 2023 Apr 5;13(4):410. doi: 10.3390/membranes13040410 (PMC10141107; doi:10.3390/membranes13040410)
Supplement: Supplementary file 1 [file membranes-13-00410-s001.zip › membranes-2300020-supplementary.pdf]

Supplementary Information

Life cycle assessment of innovative carbon dioxide selective membranes from low carbon emission sources: a comparative study

Amit S. Nilkar [1,2], Christopher J. Orme[1], John R. Klaehn, Haiyan Zhao[2] and Birendra Adhikari [1,*]

Methods

Workbook methodology

A TEA-LCA model was developed on excel by enabling macros. The TEA-LCA model has many inputs and assumptions.

Assumptions

We made several assumptions for this study. The carbon enrichment system is assumed to be single, double, or triple staged system. For the single staged system, there is one membrane unit. For the double staged system, two membrane units are in series and the permeate from the first unit is fed to the second unit. For the triple staged system, three units are in series where permeate from the first unit is fed to the second unit and the permeate from the second unit is fed to the third unit. The feed gas is 1% CO_2 and 99% N_2 by volume and it is kept constant throughout our study unless otherwise stated. For other membranes, literature values as reported in Table 1 are used as the membrane separation parameters keeping the feed gas constant as 1% CO_2 in N_2. A gas separation membrane module is assumed with the internal volume of 3 L and 1 m^2 membrane area per module and cylindrical in shape with 3:1 length to diameter ratio. Similarly, the thickness of the module is assumed to be 3.175 mm (1/8 inch). The life span of the production facility is assumed to be 30 years while the membrane replacement happens once every 5 years.

Model Inputs

Many parameters are set as variables in the model. Numbers of membrane modules, material of modules, types of membrane, performance of the membranes (CO_2/N_2 selectivity, CO_2 permeability), thickness of membranes, inlet gas composition, pressure, temperature, permeate

and retentate pressure and temperature, compression pump properties (material, efficiency, delivery pressure, delivery flowrate), vacuum pump properties (material, efficiency, delivery pressure, delivery flowrate) are the material and performance variables set in the model. Unless otherwise stated, inlet volume of feed gas of 500 MM L/h, pressure of inlet feed gas of 1 bar (14.7 psi), vacuum pressure of 0.14 bar (2 psi), temperature of inlet gas as 22 ˚C, relative humidity of 40%, 8000 hours of operation per year, CO_2 permeate factor of 0.9, permeate and retentate pressure of 1 bar each and permeate and retentate temperature of 22 ˚C each are assumed. Membrane cost, module and system material and cost, electricity cost, CO_2/N_2 selectivity, CO_2 permeability and membrane thickness are the variables used for our analysis. In this study, we have used performance parameters of different commercially available as well as literature-reported Pebax-based membranes to calculate breakeven cost of production (cost of enrichment/capture) of CO_2, as illustrated in the following table:

Table S1. Scenario inputs for the analysis

Parameter	Input values
Number of Membrane Modules	1, 2 or 3
Material of the membrane module	stainless steel, carbon steel, polypropylene, or polyethylene
CO_2/N_2 selectivity of the membrane	10-100
CO_2 permeability, Barrer	2-1000
Membrane effective thickness, µm	0.01-10
Electricity cost, US$/kWh	0.4-0.24

Mass and energy balance

Mass balance is performed on entire process. The separation is done at ambient temperature. The final product is also at ambient temperature condition. Thus, energy (heat) balance was not necessary.

Process description

The CO_2/N_2 enrichment system comprises a maximum of three membrane stages in series, Figure 3. A compressor is located before the first membrane unit. The first gas separation membrane unit follows a compressor, and a vacuum pump is installed on the permeate side of the gas separation membrane to enhance mass transport. The same units are repeated twice as

necessary making it single, double, and triple staged systems, as illustrated in Figure 2(a) of the main manuscript.

The sizing of the compressor, the first stage, and the vacuum unit depends on the amount of feed gas that needs to be processed, CO_2/N_2 selectivity and CO_2 permeability of membrane. The stages following the first module are optional as per the preferred process design. Both the compressor and vacuum pump for each membrane unit are also optional after the first stage. The second stage follows the first unit sequentially where the permeate of first becomes the feed to the second. In the model, the third stage is also considered and operated similarly.

The sizing of the pumps as well as membrane units depend on the gas volume, the pressure that needs to be generated by the pumps, any vacuum that is chosen to enhance the transmembrane driving force, and the membrane separation properties. In this work, subsequent stages are the same type of module and membrane as the first, although they do not need to be. Ultimately, the system creates a product gas with a certain purity and the model calculates the overall breakeven cost of production per kg of enriched gas product.
